# The nuclear receptor REV‐ERB *α* represses the transcription of *growth/differentiation factor 10* and *15* genes in rat endometrium stromal cells

**DOI:** 10.14814/phy2.12663

**Published:** 2016-01-26

**Authors:** Lijia Zhao, Keishiro Isayama, Huatao Chen, Nobuhiko Yamauchi, Yasufumi Shigeyoshi, Seiichi Hashimoto, Masa‐aki Hattori

**Affiliations:** ^1^ Department of Animal and Marine Bioresource SciencesGraduate School of AgricultureKyushu UniversityFukuokaJapan; ^2^ Department of Anatomy and NeurobiologyKinki University School of MedicineOsakaJapan; ^3^ Graduate School of MedicineThe University of TokyoTokyoJapan; ^4^ College of Veterinary MedicineNorthwest A & F UniversityYanglingShaanxi712100China

**Keywords:** Circadian clock, decidualization, growth/differentiation factors, REV‐ERB*α*

## Abstract

Cellular oscillators in the uterus play critical roles in the gestation processes of mammals through entraining of the clock proteins to numerous downstream genes, including growth/differentiation factor (*Gdf*)*10* and *Gdf15*. The expression of *Gdf10* and *Gdf15* is significantly increased in the uterus during decidualization, but the mechanism underlying the regulation of *Gdf* gene expression in the uterus is poorly understood. Here, we focused on the function of the cellular oscillators in the expression of *Gdf* family by using uterine endometrial stromal cells (UESCs) isolated from pregnant *Per2‐dLuc* transgenic rats. A significant decline of *Per2‐dLuc* bioluminescence activity was induced in in vitro decidualized UESCs, and concomitantly the expression of canonical clock genes was downregulated. Conversely, the expression of *Gdf10* and *Gdf15* of the *Gdf* was upregulated. In UESCs transfected with *Bmal1‐*specific siRNA, in which *Rev‐erbα* expression was downregulated, *Gdf10* and *Gdf15* were upregulated. However, *Gdf5, Gdf7*, and *Gdf11* were not significantly affected by *Bmal1* silencing. The expression of *Gdf10* and *Gdf15* was enhanced after treatment with a REV‐ERB *α* antagonist in the presence or absence of progesterone. Chromatin immunoprecipitation‐PCR analysis revealed the inhibitory effect of REV‐ERB *α* on the expression of *Gdf10* and *Gdf15* in UESCs by recognizing their gene promoters. Collectively, our findings indicate that the attenuation of REV‐ERB *α* leads to an upregulation of *Gdf10* and *Gdf15* in decidual cells, in which cellular oscillators are impaired. Our results provide novel evidence regarding the functions of cellular oscillators regulating the expression of downstream genes during the differentiation of UESCs.

## Introduction

Growth/differentiation factors (GDFs) are members of the transforming growth factor‐*β* (TGF‐*β*) superfamily, and they are involved in a variety of cellular functions and biological processes such as cell proliferation, differentiation, and remodeling (Lee 1991; McPherron et al. 1997; Whitman 1998). The expression of GDFs is involved in embryonic development and the development of female reproductive tissues. The GDFs such as GDF1, GDF3, GDF10, and GDF15 are spatiotemporally expressed in the embryo and uterus. For example, GDF10 is highly expressed in the uterus during the menstrual cycle and pregnancy (Zhao et al. 1999) and plays a role in head formation (Hino et al. 2004). GDF15, which is also known as macrophage inhibitory cytokine‐1 (MIC‐1), is highly expressed in human placenta and is thought to have a predictive aspect for pregnancy outcomes (Lawton et al. 1997; Fairlie et al. 1999; Tong et al. 2004). GDF15 is principally expressed in villous cytotrophoblast cells, extravillous trophoblasts, decidual stromal cells, and placenta (Lawton et al. 1997; Fairlie et al. 1999; Tong et al. 2004). A study using extravillous trophoblast cells demonstrated an inhibitory effect of GDF15 on cell viability by apoptosis and growth inhibition (Morrish et al. 2001). GDF15 also functions as a potent regulator of matrix metalloproteinases, which controls the degradation of the decidual matrix and thus affects the invasion of trophoblast cells (Marjono et al. 2003). However, the mechanism underlying the regulation of *Gdf* gene expression in the uterus remains poorly understood.

There are many E‐box and ROR/REV‐ERB response elements (ROREs), which are the circadian clock‐controlled *cis*‐regulatory elements, in the promoter regions of the *Gdf* genes such as *Gdf10* and *Gdf15* (NCBI Reference Sequence: NC_005115.4). Numerous peripheral circadian clocks are partially self‐operative and independent in their responses to external and internal stimuli other than the stimuli originating from the suprachiasmatic nucleus, known as the central circadian clock (Hara et al. 2001; Vollmers et al. 2009; Tahara et al. 2012; Wu et al. 2012). The molecular mechanism of the mammalian circadian clock involves a primary conservative interlocked transcriptional‐translational feedback loop (Ko and Takahashi 2006). This loop is comprised of a core group of clock genes and their protein products, which are mostly the transcription factors. The transcriptional activators BMAL1 and CLOCK form a heterodimer, which drives the expression of the *Per1‐3* and *Cry1‐2* genes by recognizing E‐box *cis*‐elements in their promoters (Gekakis et al. 1998; Hogenesch et al. 1998; Ueda et al. 2005). The CLOCK‐BMAL1 heterodimer also induces expression of the nuclear receptor, REV‐ERB*α*, resulting in the repression of the transcription of *Bmal1* through direct binding to the RORE located in the *Bmal1* promoter (Albrecht and Eichele 2003; Brown et al. 2005). In addition to regulating each other to sustain oscillations, REV‐ERB*α* also controls the expression of numerous downstream genes through binding to ROREs at their promoters.

BMAL1, a critical component of clock proteins, is indispensable in maintaining the integrity of the circadian feedback loop and the homeostasis of numerous behaviors and physiological processes (Kondratov et al. 2006; Alvarez et al. 2008; Grechez‐Cassiau et al. 2008; Ratajczak et al. 2009). Several studies provided evidence demonstrating that the physiologic significance of BMAL1 is related to mammalian reproductive functions (Ratajczak et al. 2009; Boden et al. 2010; Liu et al. 2014). REV‐ERB*α* usually functions as a transcriptional repressor for the lack of activation function (AF‐2) domain present at the C‐terminal of the ligand‐binding domain (Yin and Lazar 2005; Phelan et al. 2010; Crumbley and Burris 2011). REV‐ERB*α* recruits the endogenous nuclear receptor corepressor (N‐CoR)/histone deacetylase3 complex to repress its target gene transcription, thereby regulating a diverse array of cellular processes (Yin and Lazar 2005; Yin et al. 2006). REV‐ERB*α* was originally regarded as an orphan nuclear receptor (Miyajima et al. 1989), and thereafter heme was identified as its natural ligand (Yin et al. 2007; Meng et al. 2008; Grant et al. 2010; Kojetin et al. 2011). GSK4112 is synthesized as a chemical agonist of REV‐ERB*α*, and it represses REV‐ERB*α* target genes (Grant et al. 2010; Chen et al. 2012; Gibbs et al. 2012; Chini et al. 2013). A chemical REV‐ERB*α* antagonist, SR8278, was reported (Kojetin et al. 2011), and it increases the transcription of REV‐ERB*α* target genes (Kojetin et al. 2011; Isayama et al. 2015; Tasaki et al. 2015).

*Rev‐erbα* has a key role in several physiological actions such as adipocyte differentiation, glucose metabolism, and thermogenesis (Chawla and Lazar 1993; Cho et al. 2012; Gerhart‐Hines et al. 2013). The circadian system consisting of clock genes is also disrupted in differentiating cells of rat ovaries and uteri (Alvarez and Sehgal 2005; He et al. 2007a). Several studies have demonstrated that circadian clock genes are rhythmically expressed in the uterus (Dolatshad et al. 2006; He et al. 2007a; Hirata et al. 2009; Akiyama et al. 2010). In rodents and humans, the uterus endometrial stromal cells (UESCs) undergo proliferation and differentiation into decidual cells in response to ovarian steroids and blastocyst implantation at the early stage of pregnancy (Clarke and Sutherland 1990; Zhang et al. 1994; Dey et al. 2004). Decidualization is critical to the establishment of fetal‐maternal communication and the progression of implantation and this process ultimately results in the formation of the placenta. We observed that the *Per2* expression in UESCs is downregulated during decidualization, which influences the expression of the vascular endothelial growth factor gene (Uchikawa et al. 2011). Deregulation of the circadian clock may attenuate or disrupt the expression of clock‐controlled genes (CCGs) and can have a profound influence on organ functions. We have shown that the *Bmp* genes of the *Tgf‐β* superfamily are regulated by the attenuation of the cellular circadian clock through binding to the RORE regions of their promoters (Tasaki et al. 2015).

In light of these recent reports, we considered the possibility of cellular circadian oscillators in the regulation of the expression of *Gdf10* and *Gdf15* genes. In this study, we extended our recent investigations to examine the possible involvement of cellular circadian oscillators in the regulation of *Gdf* gene expression in stromal cells during decidualization. To do so, we used *Bmal1*‐specific small interfering (siRNA) and the REV‐ERB*α* antagonist SR8278, which attenuates or disrupts the expression of cellular circadian oscillators and can have a profound influence on the expression of CCGs. The results presented here demonstrate the regulation of REV‐ERB*α* in the transcription of *Gdf10* and *Gdf15* due to REV‐ERB*α*'s recognition of the RORE sites of their promoters.

## Materials and Methods

### Animals

All the experiments were performed under the control of the guidelines for Animal Experiments in the Faculty of Medicine, Kyushu University, and Law No. 105 and Notification No. 6 of the Government of Japan. All procedures were reviewed and approved by the Committee on the Ethics of Animal Experiments of the Kyushu University (Permit No.: A24‐054‐2). *Per2‐dLuc* transgenic rats were obtained from our breeding colony. In this transgenic rat, the mouse *Per2* promoter region, which is sufficient for circadian oscillation, was fused to a *dLuc* reporter gene (Ueda et al. 2005). The transgenic rats were maintained under a 12:12‐h light–dark cycle (zeitgeber time, ZT0: 0800 light on; ZT12: 2000 light off) and ad libitum feeding throughout all experiments.

### Preparation of total RNA from uterine tissues

Uterine tissues were collected at 2000 h (ZT12) on days 4.5 (D4.5) and 6.5 of gestation. Uterine tissues from day 6.5 rats were cut into pieces carefully at interimplantation site and implantation site. Samples from the implantation site contained the whole uterine components and embryos. Pieces of uterine tissues (ca. 20 mg) were homogenized in 1 mL of lysis buffer (Qiagen, Hilden, Germany) with a disposable homogenizer (BioMasher, Funakoshi, Tokyo). During homogenization, the perimetric tissues were removed. Total RNA was isolated using an RNeasy Mini kit (Qiagen) and cDNAs were generated by RT with Oligo (dT)_15_ and Random Primers according to the manufacture's protocol. The RNA concentration was determined using 260/280‐nm spectrophotometry (Pharmacia Biotech, Buckinghamshire, UK), and RNA integrity was checked by agarose gel electrophoresis.

### Preparation and culture of UESCs

The UESCs were isolated from *Per2‐dLuc* transgenic rats on day 4.50 (D4.50) of gestation as reported previously (He et al. 2007a; Oozono et al. 2008; Matsumoto et al. 2009). The uterine lumens were filled with PBS containing 0.1% collagenase (Invitrogen, Carlsbad, CA) and incubated at 37°C for 1 h in a shaking water bath. The harvested cells were washed thrice with fresh DMEM/F12 (Invitrogen), and seeded onto 35 mm collagen‐coated dishes (Iwaki, Tokyo) at the density of 2 × 10^5^ cells/dish with 2 mL of culture medium [phenol red‐free DMEM/F12 supplemented with 10% charcoal‐treated FBS (Invitrogen) and 1× PS (Nacalai Tesque, Kyoto)]. The culture medium was replaced at 15 min after cell seeding to remove epithelial cells. Cells were cultured in a humidified atmosphere of 5% CO_2_ at 37°C for 2 days. Then, cells were cultured in serum‐free medium supplemented with 1× antibiotic–antimycotic (AA; Nacalai Tesque), 1× Insulin‐Transferrin‐Selenium (ITS, Life Technologies, Grand Island, NY), 0.1% bovine serum albumin (BSA, Sigma‐Aldrich, St Lousis, MO), and 100 nmol/L progesterone (P_4_, Sigma‐Aldrich) for additional 2 days prior to other treatments.

### In vitro decidualization

Confluent UESCs were further cultured for 5 days in DMEM/F12 supplemented with 0.1 mmol/L medroxyprogesterone acetate (MPA, Sigma‐Aldrich), 0.5 mmol/L 2‐O‐dibutyryl cAMP (db‐cAMP, Sigma‐Aldrich), 1× AA, 1× ITS, 0.1% BSA, as previously described (Matsumoto et al. 2009). The differentiating status was revealed by the expression of the *Prl8a2* gene (Jabbour and Critchley 2001).

### Real‐time monitoring of Per2‐dLuc oscillations

The cultured UESCs were synchronized with 100 nmol/L dexamethasone (DXM, Sigma‐Aldrich) for 2 h in the serum‐free medium containing 1× AA. Then, cells were given the serum‐free medium DMEM/F12 supplemented with 0.1 mmol/L luciferin (Wako, Tokyo), 0.1% BSA, 1× AA, and 1× ITS, and subjected to luminescence determination. Luciferase activity was monitored at 37°C with a Kronos Dio AB‐2550 luminometer (ATTO, Tokyo) interfaced to a computer for continuous data acquisition (He et al. 2007b; Hirata et al. 2009). In some experiments, confluent UESCs were synchronized with DXM, and then monitoring was performed in the presence of 10 μmol/L SR8278 (Sigma‐Aldrich) or 0.1% DMSO (vehicle control). The data are presented as photon counts per min. Bioluminescence data were detrended by subtracting the 24‐h running average from the raw data. Detrended datasets were smoothed by taking 2‐h running averages. The amplitude and period of *Per2‐dLuc* oscillations were documented by the single Cosinor method using Timing Series Single 6.3 (Expert Soft Tech., Richelieu, France).

### Microarray analysis

RNA samples isolated from cultured UESCs at 30, 36, 42, and 48 h after synchronization with DXM were used for microarray analysis using the Whole Rat Genome Microarray 4x44K Ver3.0 (Agilent Technologies, Santa Clara, CA) representing 30,367 probe sets. Bioinformatics analysis was performed using Agilent Future Extraction software (Agilent Technologies). The data were filtered for signal intensity values (*P *<* *0.05, detectable), which allowed removing very low signal values (Tasaki et al. 2013). The ratio of signal intensity values was calculated for the *Gdf* gene family.

### Bmal1 siRNA transfection

RNA oligos targeting the *Bmal1* mRNA (F: GAAUGUCACAGGCAAGUUUdTdT; R: AAACUUGCCUGUGUGACAUUCdTdT) and nonsilencing RNA (mission_SIG‐001) were purchased from Sigma‐Aldrich. Cultured UESCs were first plated in 35‐mm collagen‐coated dishes with 2 mL DMEM/F12 supplemented with 1× AA, 1× ITS, 0.1% BSA. The medium was removed after 24 h, and the cells were transfected with the RNA oligos diluted in Opti‐MEM using Lipofectamine^®^ RNAiMAX reagent (Life Technologies) according to the manufacturer's protocol (Chen et al. 2013a). Both the *Bmal1*‐specific siRNA and nonsilencing RNA were used at a final concentration of 25 nmol/L. The cells were maintained with transfection medium for duration of 12 h. Then the medium was replaced with DMEM/F12 supplemented with 1× AA, 1× ITS, 0.1% BSA, and 100 nmol/L P_4_. After 48 h in culture, cells were synchronized with DXM for monitoring of luciferase activity.

### RNA extraction and RT‐PCR

Uterus tissues were collected from pregnant rats on D4.50 (ZT4) of gestation. The pieces of uterine tissues (0.1 g) were homogenized in 1 ml of Sepasol‐RNA I Super (Nacalai Tesque) for 2 min (Uchikawa et al. 2011), and total RNA was isolated using an RNeasy Mini kit (Qiagen) according to the manufacturer's protocol. RNA samples were treated with RNase‐free DNase (Qiagen). The cDNAs were generated by RT with Oligo (dT)_15_ and Random Primers using a GoTaq^®^ 2‐Step RT‐qPCR System (Promega). The PCR reaction was performed in 10 *μ*L of 1× PCR buffer, 0.2 mmol/L each of dNTP, 0.25 U AmpliTaq Gold (Applied Biosystems), 0.2 μmol/L each of the synthetic primer sets (Table 1), and 20 ng cDNA. The amplification was performed in 42 cycles and resulting PCR products were analyzed by electrophoresis on 1.8% agarose gels.

**Table 1:** Primer sequences for the targeted genes in real‐time qPCR and RT‐PCR

Gene	Accession No.	Sequence 5′–3′	Amplicon (bp)
*Bmal1*	NC_005100	F: CCGTGGACCAAGGAAGTAGA	97
		R: CTGTGAGCTGTGGGAAGGTT	
*Rev‐erbα*	NM_031134	F: ACAGCTGACACCACCCAGATC	102
		R: CATGGGCATAGGTGAAGATTTCT	
*Per2*	NM_031678	F: GACGGGTCGAGCAAAGGA	90
		R: CCCTTTTCAGGTGTATAGGTAAGT	
*Dbp*	NM_012543	F: GCAAGGAAAGTCCAGGTGCCCG	95
		R: GCGTCTCTCGACCTCTTGGCT	
*Gdf5*	XM_001066344	F: ATCTTTAGGCCAGGGGGTCA	143
		R: GGTCCTGGCTTGGTTTCAGA	
*Gdf7*	NM_001170350	F: TCACAGACCAAGCAACTGAAG	98
		R: ATTCACCACCTCGTGGGAG	
*Gdf10*	NM_024375	F: CCTACTACTGTGCTGGAGCC	75
		R: TCTGGATGGTGGCATGGTTG	
*Gdf11*	XM_343148	F: GGGCAAGAGGGCTAACACAT	102
		R: TCTGAACTGCTTCCGTGAAC	
*Gdf15*	NM_019216	F: CCAGCTGTCCGGATACTCAG	106
		R: GGTAGGCTTCGGGGAGACC	
*Prl8a2*	NM_017008	F: ATCCAGCGAGCTGAAGTCAT	178
		R: CATGAAGTGGTGGGTTTGTG	
*Gapdh*	NM_017008	F: ATGGCCTTCCGTGTTCCTAC	122
		R: CTTTACAAAGTTGTCGTTGA	

### RT‐qPCR

Cultured cells were harvested at indicated time points. Total RNA was isolated using an RNeasy Mini kit (Qiagen) and cDNAs were generated by RT with Oligo (dT)_15_ and Random Primers as described above. RT‐qPCR was performed in a 50‐*μ*L volume containing a 20‐ng cDNA sample in GoTaq^®^ qPCR Master Mix and 250 nmol/L specific primers listed in Table 1, with the Mx3000P Real‐time qPCR System (Agilent Technologies, Santa Clara, CA) using the parameters as described in our previous report (Chen et al. 2013b). All reactions were performed in triplicate and displayed amplification efficiency between 80% and 120%. Relative quantification of each mRNA was performed using the comparative quantity (copies) method creating standard curves. The quantity for each sample was normalized to *Gapdh*.

### Chromatin immunoprecipitation (ChIP assay)

Confluent UESCs were synchronized with DXM and then harvested at 48 h and 3.0 × 10^6^ cells were used per one IP reaction. ChIP assay was performed by using SimpleChIP Plus enzymatic chromatin IP kit (Cell Signaling Technology, Beverly, MA), as instructed by the manufacturer's protocol. Briefly, cells were fixed with 1.0% formaldehyde for 10 min to cross‐link proteins to DNA, and the cross‐linking was stopped with glycine. The extracted genomic DNA was digested with 0.5 *μ*L micrococcal nuclease for 20 min. After centrifugation at 16,500 *g* for 1 min, the nuclear pellet was suspended in the ChIP buffer containing protease inhibitors and lysed with sonication (3 pulses, 20 sec). After determining DNA concentration, the cross‐linked chromatin (2 *μ*g) was incubated overnight at 4°C with an REV‐ERB*α* antibody (5 *μ*g/500 *μ*L; Cell Signaling Technology) as the positive control and normal rabbit IgG (1 *μ*g/500 *μ*L) as the negative control and incubated with Protein G agarose beads for additional 2 h. The chromatin was eluted from the agarose beads by incubating for 30 min at 65°C and the cross‐links were reversed by incubating with 5 mol/L NaCl (6 *μ*L/150 *μ*L) and proteinase K (2 *μ*L/150 *μ*L) for 2 h at 65°C. Purified DNA was amplified by PCR with specific primer sets as follows: *Gdf10*: forward 5′‐GCTTGCACAGATTGCTTCTTGT‐3′ (‐2702/‐2681) and reverse 5′‐GCCTCATTTTACTGCCGAACAG‐3′ (‐2526/‐2505): (NC_005115.4); *Gdf15*: forward 5′‐TCTACAGGAGGAGGGGGACTA‐3′ (‐1163/‐1143) and reverse 5′‐GCCAGGTAGGTGCATGGTAAG‐3′ (‐1002/‐982) (NC_005115.4). The amplification was performed in 35 cycles and PCR products were analyzed by electrophoresis on 1.8% agarose gels.

### Statistical analyses

All data are expressed as means ± S.E.M. of at least three separate experiments, each performed with duplicate samples. The amplitude of *Per2‐dLuc* was determined by the single Cosinor method using Timing Series Single 6.3 (Expert Soft Tech.). The statistical differences in examined values were determined by one‐way or two‐way ANOVA followed by a Bonferroni's post hoc test using SigmaPlot software (Ver. 11.2; Systat Software, San Jose, CA). Differences were considered significant at *P *<* *0.05 or less.

## Results

### Expression levels of the *Gdf* genes in cultured UESCs by microassay

**Figure 1:**
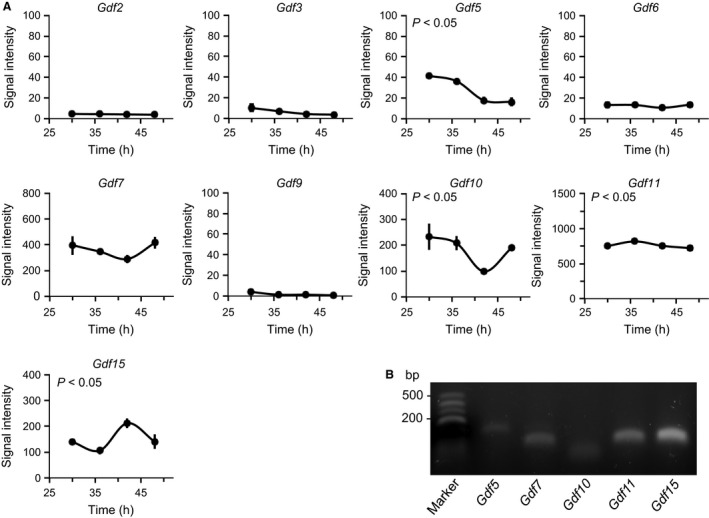
Expression profiles of the *Gdf* genes in UESCs isolated from pregnant rats as determined by DNA microarray and RT‐PCR analyses of core *Gdf* genes. (A) According to the indicated protocols, RNA samples isolated from cultured UESCs and subjected to microarray analysis. Each value represents the means ± S.E. (*n* = 3) of signal intensity from the microarray results. (B) Total RNA was isolated from UESCs and subjected to RT‐PCR for core *Gdf* genes (*Gdf5*,* Gdf7*,* Gdf10*,* Gdf11*, and *Gdf15*) with specific primer sets.

To gain insight into the cellular clocks of UESCs and the expression of the *Gdf* genes, we analyzed the global expression of the *Gdf* genes by performing DNA microarrays (Tasaki et al. 2013). The expression levels of *Gdf5, Gdf7, Gdf10, Gdf11, and Gdf15*, designated as core *Gdf* genes, were relatively high compared to those of *Gdf2, Gdf3, Gdf6, and Gdf9* (Fig. 1A). Of the core *Gdf* genes, the alterations of *Gdf5*,* Gdf10*,* Gdf11,* and *Gdf15* transcript levels were significant (*P *<* *0.05). We also confirmed the detection of the transcripts of core *Gdf* genes in UESCs by RT‐PCR (Fig. 1B). The core *Gdf* genes were analyzed in the following experiments.

### Expression levels of core *Gdf* genes in whole‐uterus tissues during decidualization

**Figure 2:**
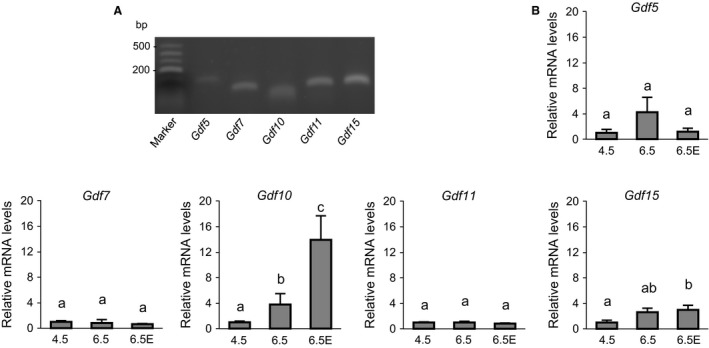
Expression levels of core *Gdf genes* in pregnant rat uterus during implantation and decidualization. (A) Total RNA samples were collected from the uterus of pregnant rats on pregnant day 4.5 and subjected to RT‐PCR for the expression levels of core *Gdf* genes. (B) Total RNA samples were collected from the uterus of pregnant rats on pregnant day 4.5 (implantation) or ZT12 (D4.5) and D6.5 (decidualization) or ZT12 (D6.5) and subjected to RT‐qPCR. The D6.5 samples were divided into implantation sites (D6.5E) and inter‐implantation sites (D6.5). The transcript levels were calculated and normalized to each value given by the D4.5 sample. Values with different letters are significantly different (*P *<* *0.05).

To further analyze the expression of core *Gdf* genes in the early pregnancy period, we measured the expression levels of *Gdf5, Gdf7, Gdf10, Gdf11, and Gdf15* in the whole uterus of pregnant rats on D4.5, defined as the implantation period, by RT‐PCR. As shown in Figure 2A, the transcripts of all core *Gdf* genes were detected. To investigate the temporal changes in *Gdf* genes during the implantation and decidualization periods, we analyzed the transcript levels of *Gdf* genes in the rat uterus on D4.5, at interimplantation sites (D6.5), and at implantation sites on day 6.5 (D6.5E). As shown in Figure 2B, the transcript level of *Gdf10* was significantly increased on D6.5 compared to that on D4.5 (*P *<* *0.05). The transcript levels of *Gdf10* and *Gdf15* were significantly higher at implantation sites on D6.5E than those on D4.5 (*P *<* *0.05). Conversely, the expression levels of *Gdf5*,* Gdf7*, and *Gdf11* in the pregnant rat uteri were not significantly altered during the implantation or decidualization periods (Fig. 2B).

### Downregulation of *Per2‐dLuc* oscillation and canonical clock gene expression in decidual cells

**Figure 3:**
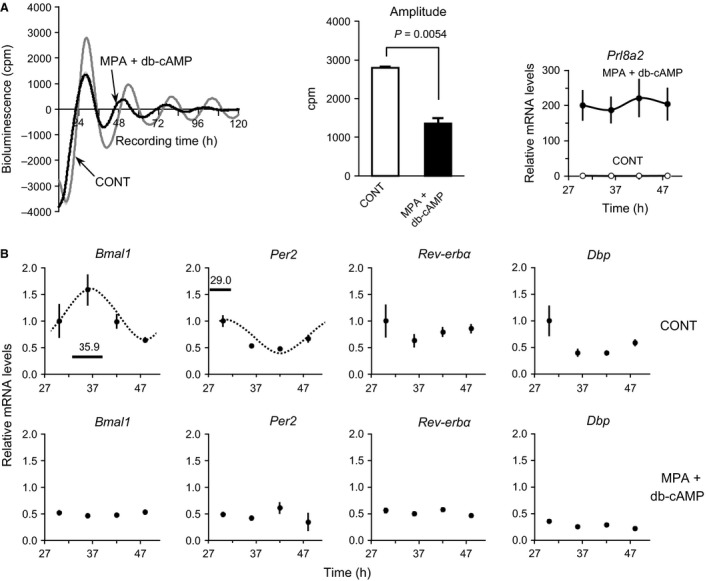
*Per2* oscillation profiles and expression levels of core clock genes in UESCs during in vitro decidualization. (A) UESCs were isolated from the uterine horns on day 4.5 of pregnancy and cultured for 3 days prior to any treatment. Decidual cells were obtained by treating confluent UESCs for 4.5 days with 0.1 mmol/L MPA plus 0.5 mmol/L db‐cAMP. Both types of cells were synchronized with DXM for 2 h prior to monitoring. The amplitude of *Per2‐dLuc* oscillations was determined by the single Cosinor method. The mRNA level of *Prl8a2*, a marker for UESCs decidualization, was analyzed in the control and decidual cells by RT‐qPCR. (B) According to the first *Per2‐dLuc* phase in *panel A*, total RNA samples were collected from control and decidual cells at the indicated times after synchronization and RT‐qPCR analyses of transcript levels were performed. The transcript levels were calculated and normalized to each value given by the control sample at 30 h. The Cosinor analysis method was used to determine the rhythmic expression of examined genes. Statistical rhythmicity (*dotted curves*,* P *<* *0.05) and peak time with 95% confidential intervals (*line length*) are shown in each panel.

To further investigate whether decidualization induces changes in the cellular clockwork, we used MPA and db‐cAMP to induce the in vitro decidualization of UESCs prepared from pregnant rats on D4.5. After the control and decidualized UESCs were synchronized with 100 nmol/L DXM for 2 h, luciferase activity was chronologically monitored. Both cell groups generated several *Per2‐dLuc* oscillations, but a significant attenuation of *Per2‐dLuc* bioluminescence was observed in the decidual cells (*P *=* *0.0054, vs. CONT) (Fig. 3A). A significant increase in the transcript level of *Prl8a2*, a decidualization marker, was observed in the treated cells (two‐way ANOVA, *P *<* *0.001). Circadian rhythms of *Bmal1*,* Per2, Rev‐erbα,* and *Dbp* were not detected in the decidual cells, in which the transcript levels of these genes were significantly reduced compared to the levels in the control cells (two‐way ANOVA, *P *<* *0.01). Thus, the expression of canonical clock genes was attenuated in decidual cells.

### Expression levels of core *Gdf* genes in decidual cells

**Figure 4:**
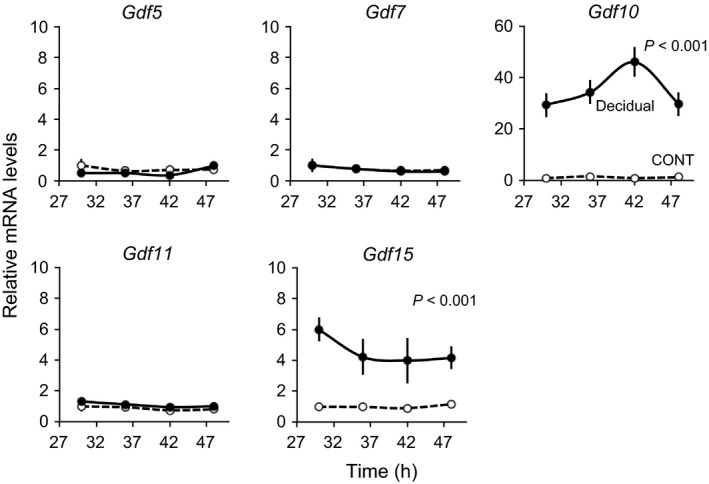
Expression profiles of core *Gdf* genes in decidual cells. According to the first *Per2‐dLuc* phase shown in Figure 3A, total RNA samples were collected from control (*dot line*) and decidual (*solid line*) cells at the indicated times after synchronization. RT‐qPCR analyses of transcript levels were performed using their specific primers for *Gdf5*,* Gdf7*,* Gdf10*,* Gdf11*, and *Gdf15*. The transcript levels were calculated and normalized to each value given by the control sample at 30 h.

We analyzed the expression of *Gdf5, Gdf7, Gdf10, Gdf11, and Gdf15* in decidual cells induced by MPA and db‐cAMP. The transcripts of *Gdf10* and *Gdf15* were increased in the decidual cells by more than 30‐fold and fourfold, respectively (two‐way ANOVA, *P *<* *0.001, Fig. 4). Conversely, the transcript levels of *Gdf5, Gdf7,* and *Gdf11* were not significantly altered in the decidual cells.

### Effects of *Bmal1* siRNA treatment on the expression of canonical clock genes and *Gdf* genes in UESCs

**Figure 5:**
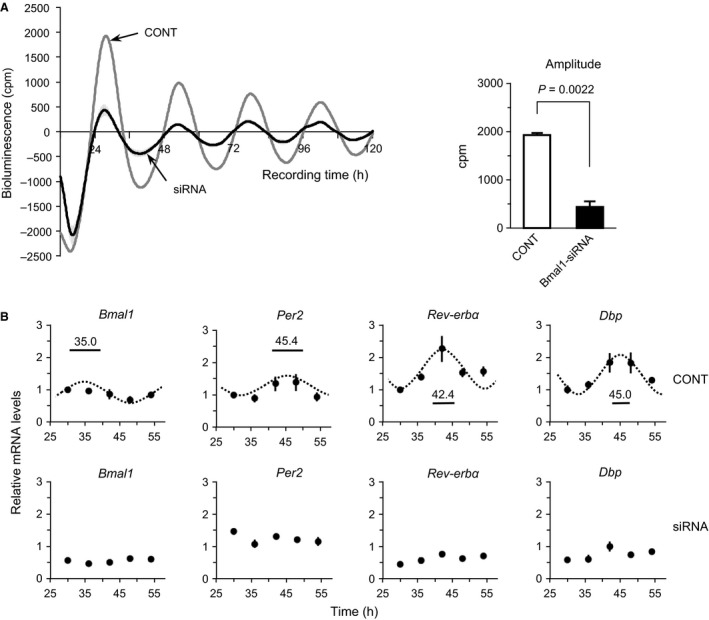
Effect of *Bmal1*‐specific siRNA treatment on *Per2‐dLuc* oscillations in UESCs. (A) Representative records of bioluminescence showing the effect of *Bmal1* interference on the rhythmic expression of *Per2‐dLuc* oscillations in UESCs. UESCs were treated with *Bmal1*‐specific siRNA (Bmal1‐siRNA) or nonsilencing RNA (CONT) according to the indicated protocols. The cells were then synchronized with DXM for bioluminescence determination (time: 0 h). Amplitude of *Per2‐dLuc* oscillation in UESCs was estimated with or without *Bmal1* siRNA treatment. (B) According to the first *Per2‐dLuc* phase (*solid line*) in *panel A*, total RNA samples were collected from control and siRNA‐treated cells at the indicated times after synchronization. RT‐qPCR analyses of transcript levels were performed using their specific primers. The transcript levels were calculated and normalized to each value given by the control sample at 30 h. The Cosinor analysis method was used to determine the rhythmic expression of examined genes. Statistical rhythmicity (*dotted curves*,* P *<* *0.05) and peak time with 95% confidential intervals (*line length*) are shown in each panel.

**Figure 6:**
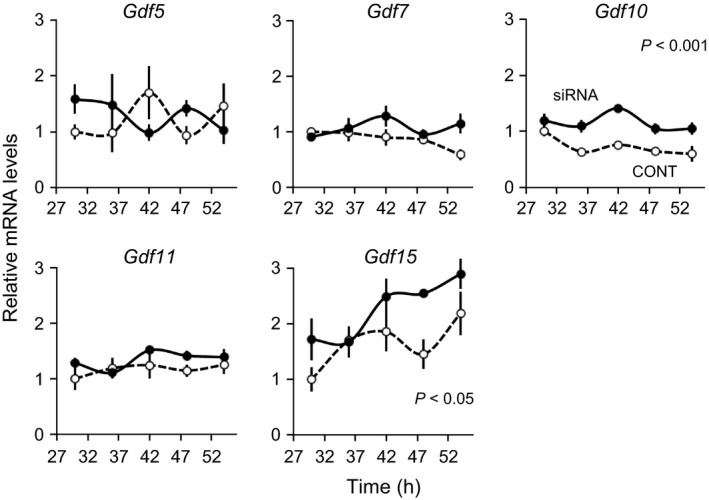
Expression profiles of core *Gdf* genes in UESCs transfected with *Bmal1*‐specific siRNA or nonsilencing RNA. According to the first *Per2‐dLuc* phase shown in Figure 5A, total RNA samples were collected from control (*dot line*) and decidual (*solid line*) cells at the indicated times after synchronization. RT‐qPCR analyses of transcript levels were performed using their specific primers for *Gdf5*,* Gdf7*,* Gdf10*,* Gdf11*, and *Gdf15*. The transcript levels were calculated and normalized to each value given by the control sample at 30 h.

To analyze whether the cellular circadian clock in UESCs contributes to the expression of *Gdf* genes, we transfected *Bmal1*‐specific siRNA (siRNA) into UESCs. Given the critical role of *Bmal1* in the sustaining of the cellular circadian rhythm, we investigated *Per2‐dLuc* oscillations using UESCs transfected with siRNA or nonsilencing RNA (CONT). Both cell groups generated several *Per2‐dLuc* oscillations (Fig. 5A). However, the amplitude of *Per2‐dLuc* bioluminescence was significantly reduced in the siRNA‐treated cells (*P *=* *0.0022). The circadian rhythm of *Bmal1* was not detected after the siRNA transfection (Fig. 5B). Concomitantly, the circadian rhythms at the transcript levels of *Per2*,* Rev‐erbα*, and *Dbp*, which are downstream genes of *Bmal1*, were attenuated in the siRNA‐treated cells. The transcripts of *Gdf10* and *Gdf15* were significantly increased in the siRNA‐treated cells (two‐way ANOVA, *P *<* *0.001 and *P *<* *0.05, respectively) (Fig. 6). No significant alterations in *Gdf5, Gdf7,* and *Gdf11* transcript levels were observed in the siRNA‐treated cells. Thus, the expression of *Gdf10* and *Gdf15*, but not those of *Gdf5, Gdf7,* and *Gdf11*, was upregulated by attenuation of the cellular circadian clocks.

### Effect of SR8278 treatment on the expression of *Gdf10* and *Gdf15* in UESCs

**Figure 7:**
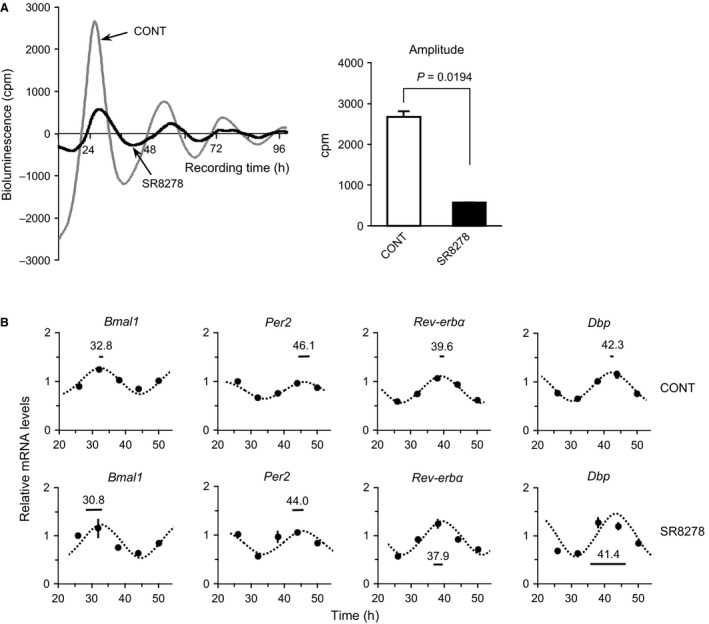
Effect of the REV‐ERB *α* antagonist SR8278 treatment on *Per2‐dLuc* oscillations and clock genes expression in UESCs. (A) Representative records of bioluminescence showing the effect of SR8278 on the rhythmic expression of *Per2‐dLuc* oscillations in UESCs. Cultured UESCs were washed twice with serum‐free medium and subjected to *Per2‐dLuc* bioluminescence determination with 10 μmol/L SR8278 or 0.1% DMSO (vehicle control) (time: 0 h). Amplitude of *Per2‐dLuc* oscillation was estimated in the cells with or without SR8278 treatment. (B) According to the first *Per2‐dLuc* phase, total RNA samples were collected from control and SR8278‐treated cells at the indicated times after synchronization. RT‐qPCR analyses of transcript levels were performed using their specific primers. The transcript levels were calculated and normalized to each value given by the control sample at 26 h. The Cosinor analysis method was used to determine the rhythmic expression of examined genes. Statistical rhythmicity (*dotted curves*,* P *<* *0.05) and peak time with 95% confidential intervals (*line length*) are shown in each panel.

**Figure 8:**
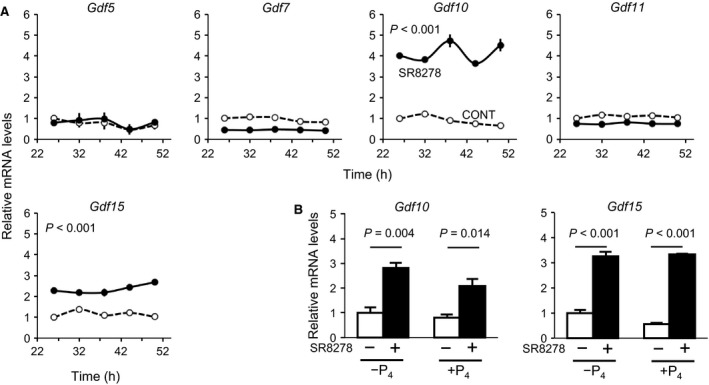
Expression profiles of core *Gdf genes* in UESCs treated with the REV‐ERB *α* antagonist SR8278. (A) According to the first *Per2‐dLuc* phase shown in Figure 7A, total RNA samples were collected from control and SR8278‐treated cells at the indicated times after synchronization. RT‐qPCR analyses of transcript levels were performed using their specific primers. Effect of SR8278 treatment on the expression of *Gdf10* and *Gdf15* was also performed in the presence or absence of P_4_. The transcript levels were calculated and normalized to each value given by the control sample at 26 h.

To further understand the physiological function of the UESC circadian clock and to detect whether *Gdf10* and *Gdf15* are controlled under the regulation of REV‐ERB*α*, we treated UESCs with the REV‐ERB*α* antagonist SR8278. A significant decline of *Per2‐dLuc* bioluminescence oscillation amplitude was observed in the SR8278‐treated cells (*P *=* *0.0194, vs. CONT) (Fig. 7A). However, the circadian rhythms of the canonical clock genes were not significantly altered by the SR8278 treatment (Fig. 7B). We observed that the transcripts of *Gdf10* and *Gdf15* were significantly increased in the SR8278‐treated cells (*P *<* *0.001, vs. CONT) (Fig. 8). Conversely, the transcript levels of *Gdf5, Gdf7,* and *Gdf11* were not upregulated by SR8278 treatment. Regardless of the presence or absence of P_4_, the SR8278 treatment caused significant increases in the expression levels of *Gdf10* and *Gdf15*.

### REV‐ERB*α* binding to the putative RORE sites in the upstream regions from transcriptional start sites of *Gdf10* and *Gdf15*

**Figure 9:**
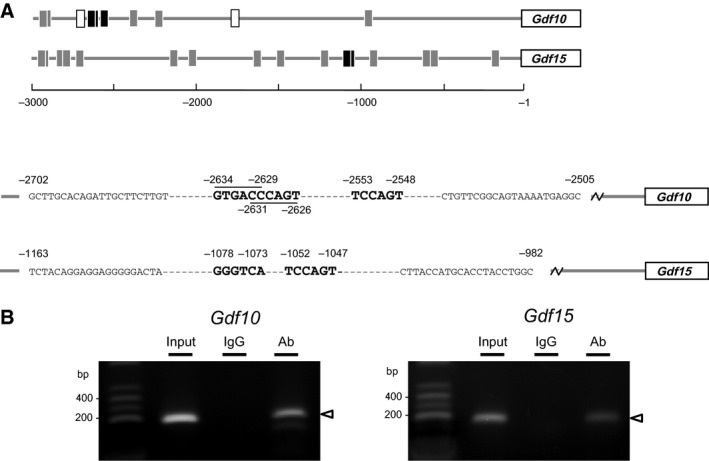
ChIP‐PCR analysis of the putative RORE sites in upstream regions from transcriptional start sites of *Gdf10* and *Gdf15*. (A) The putative RORE (*black and gray squares*) and E‐box sites (*open squares*) located in upstream regions from transcriptional start sites of *Gdf10* and *Gdf15* are listed, and distal and proximal sites were analyzed, respectively (*black squares*). Three and two putative RORE sites (*gothic letters*), GTGACC ^−2634/−2629^, CCCAGT ^−2631/−2626^, and TCCAGT ^−2553/−2548^ for *Gdf10* and GGGTCA ^−1078/−1073^ and TCCAGT ^−1052/−1047^ for *Gdf15*, located in upstream regions from transcriptional start sites of *Gdf10* and *Gdf15* were targeted, respectively. Purified DNA was amplified by PCR with specific primer sequences (*small letters*) (B) Confluent UESCs (3.8 × 10^6^ cells) were synchronized with DXM, and then harvested at 48 h and fixed with formaldehyde to cross‐link proteins to DNA as described in Materials and Methods. The extracted genome DNA was digested with Micrococcal Nuclease into fragments. After DNA purification, the cross‐linked chromatin (2 *μ*g) was used for immunoprecipitation using REV‐ERB *α* antibody and normal rabbit IgG (negative control). Purified DNA was amplified with specific primer sets for *Gdf10* and *Gdf15*. Input, input sample; IgG, negative control; Ab, anti‐REV‐ERB *α* antibody.

To further investigate whether REV‐ERB*α* exerts an inhibitory effect on the expression of *Gdf10* and *Gdf15* through direct binding to the RORE sites, we performed a ChIP‐PCR analysis. We searched putative RORE sites within the 3000‐bp upstream regions from the transcriptional start sites of *Gdf10* and *Gdf15*. Many putative RORE sites are located in these regions. Of these putative RORE sites, we selected several sites with a mass and short distance (less than 200 bp) at distal or proximal regions (Fig. 9A). We prepared the protein/DNA cross‐linked chromatin and immunoprecipitated it with an anti‐REV‐ERB*α* antibody and normal rabbit IgG (negative control). The results of this ChIP‐PCR analysis revealed specific bands detected in both the *Gdf10* and *Gdf15* samples precipitated with the antibody (Fig. 9B). These results indicated that REV‐ERB*α* directly bound to at least GTGACC^−2634/−2629^, CCCAGT^−2631/−2626^, and TCCAGT^−2553/−2548^ in the *Gdf10* promoter and GGGTCA^−1078/−1073^ and TCCAGT^−1052/−1047^ in the *Gdf15* promoter.

## Discussion

Circadian oscillators are expressed in various peripheral tissues including the ovary and uterus and may play critical roles in the regulation of reproductive physiological processes (Dolatshad et al. 2006; He et al. 2007a; Hirata et al. 2009; Akiyama et al. 2010; Uchikawa et al. 2011). To address these possible roles, we focused on the function of the circadian oscillators in the expression of *Gdf* family members by using rat UESCs. The results indicate that a few *Gdf* family members are repressed by the binding of REV‐ERB*α*, a canonical clock component, to the RORE sites located at *Gdf* putative promoters. We found that the expression levels of *Gdf10* and *Gdf15* were significantly increased in the embryo implantation sites of the pregnant D6.5 rat uterus compared to that at pregnant D4.5. We and other research group also observed that the canonical clock genes *Bmal1*,* Rev‐erbα*, and *Per2* were downregulated in UESCs during the in vitro decidualization (Isayama et al. 2015; Muter et al. 2015; Tasaki et al. 2015).

These results are consistent with our previous observations of *Per2* protein levels using whole‐uterus tissues (Uchikawa et al. 2011). The expression levels of *Gdf10* and *Gdf15* were significantly increased in the decidual cells, and the knockdown of *Bmal1*‐specific mRNA induced significant increases in the expression levels of *Gdf10* and *Gdf15*. We also found that *Gdf10* and *Gdf15* were upregulated by the inhibition of REV‐ERB*α* function by using its antagonist SR8278. However, the expression of *Gdf10* and *Gdf15* was not altered regardless of the presence or absence of P_4_, suggesting that REV‐ERB*α* does not coordinate with P_4_ in the expression of these genes. After validating the RORE sites, we assessed the inhibitory effect of REV‐ERB*α* on the expression of *Gdf10* and *Gdf15* in the uterus prior to embryo implantation.

Previous reports implicated the *Gdf* genes in the regulation of prenatal uterine development (Lawton et al. 1997; Zhao et al. 1999; Tong et al. 2004), and *Gdf* family members were demonstrated to play critical roles in the regulation of oocyte development (Gui and Joyce 2005). Analyses using DNA microarrays, RT‐qPCR, and RT‐PCR revealed the expression of *Gdf5*,* Gdf7, Gdf10, Gdf11,* and *Gdf15* in the UESCs of pregnant D4.5 rats (Figs. 1, 2). Of these core *Gdf* genes, it is noted that in this study the transcripts of *Gdf10* and *Gdf15* were also increased in the uterus on D6.5, in particular at the implantation site (D6.5E), compared to D4.5, suggesting roles of *Gdf10* and *Gdf15* in embryo implantation (Fig. 2). These results may support the contention that *Gdf10* and *Gdf15* are critical for the formation of placenta (Zhao et al.^1999^; Moore et al. 2000; Segerer et al. 2012).

In rodents and humans, uterine receptivity implies a dialog between UESCs and free‐floating blastocysts. The UESCs undergo proliferation and differentiation into decidual cells, and the placenta is ultimately formed. In this study, the decidual cells induced by the in vitro treatment with MPA and db‐cAMP displayed attenuation of the rhythmic expression of canonical clock genes as well as the generation of *Per2‐dLuc* oscillations (Fig. 3). Consistently, our results demonstrated that the canonical clock genes were significantly downregulated in the decidual cells and lost rhythmic expression. The underlying mechanism of attenuated clockwork in decidual cells is not yet clear. One possibility is that regular interaction of clock proteins and chromosomal DNA is disrupted or impaired at the level of gene transcription due to the requirement of differentiation‐specific gene transcription. Especially, BMAL1 is indispensable in maintaining the integrity of the circadian feedback loop and the homeostasis of numerous physiological states (Rudic et al. 2004; Shimba et al. 2005). Recent our studies also indicate the downregulation of *Bmal1* in in vivo and in vitro experiments (Isayama et al. 2015; Tasaki et al. 2015). However, differentiation‐inducing factor(s) in UESCs are not identified yet.

However, the relationship between the circadian clockwork and the *Gdf* gene expression during the differentiation of the UESCs into the decidual cells remains largely undefined. We suggested that some of the *Gdf* genes are under the direct regulation of uterine circadian oscillators. To investigate this hypothesis, we examined highly expressed *Gdf* genes such as *Gdf5*,* Gdf7*,* Gdf10*,* Gdf11*, and *Gdf15* from the DNA microarray (Fig. 1) and measured the transcript levels of these core *Gdf* genes in the UESCs of pregnant D4.5 rats. The results indicate that the *Gdf* genes are differentially regulated in the UESCs.

It is well established that *Bmal1* is indispensable in sustaining the transcriptional‐translational feedback loop. In this study using *Bmal1*‐specific siRNA, the expression of the core clock genes *Per2*,* Rev‐erbα,* and *Dbp* was significantly reduced, as was the amplitude of *Per2‐dLuc* oscillations (Fig. 5). These results are similar to those of an earlier investigation in which the levels of *Per1*,* Per2*,* Rev‐erbα,* and *Dbp* mRNA were low in *Bmal1* null mice (Bunger et al. 2000; Boden et al. 2010). Our findings indicate that the expression of clock genes is impaired in *Bmal1* siRNA‐treated UESCs, providing evidence for further studies of CCGs in the uterus.

Interestingly, the *Bmal1* silencing induced significant increases in *Gdf10* and *Gdf15* mRNA levels, but it did not affect the expression of *Gdf5*,* Gdf7*, or *Gdf11* (Fig. 6). Because of the presence of clock‐controlled *cis*‐regulatory elements within their promoters and their rhythmic expression, the present results suggest that these genes are directly under the regulation of canonical clock genes.

REV‐ERB*α* is an important nuclear hormone receptor (NHR) in the regulation of cell physiology. Substantial evidence has shown that REV‐ERB*α* is a circadian clock component in the maintenance of circadian rhythms (Preitner et al. 2002). SR8278, a synthetic antagonist of REV‐ERB*α*, can inhibit the activity of REV‐ERB*α* to increase the expression of its target genes (Kojetin et al. 2011; De et al. 2015). In this study, we examined the expression of *Gdf10* and *Gdf15* in cultured UESCs using the antagonist SR8278. The addition of SR8278 increased the *Gdf10* and *Gdf15* transcript levels due to the decreased activity of REV‐ERB*α* (Fig. 8). REV‐ERB*α* is recruited to the target gene promoters by its binding to the agonist heme (Raghuram et al. 2007). In the presence of SR8278, this increase in *Gdf10* and *Gdf15* transcript levels may be the result of a competitive inhibition of REV‐ERB*α* binding to heme. Intriguingly, we observed that the increased transcriptions of *Gdf10* and *Gdf15* in cultured UESCs treated with SR8278 were not affected in the presence of P_4_ (Fig. 6). Thus, P_4_ is not related to the expression of *Gdf10* or *Gdf15* in UESCs. Interestingly, *Gdf5*,* Gdf7*, and *Gdf11* did not display upregulation after SR8278 treatment, similar to those observed in the pregnant uteri during the implantation or decidualization periods. These results conclude that the expression of *Gdf5*,* Gdf7*, and *Gdf11* is independent of circadian clockwork in UESCs.

The present results might indicate that REV‐ERB*α* has an inhibitory effect on the regulation of *Gdf10* and *Gdf15* expression in UESCs of pregnant D4.5 rats. It is interesting to note that a mass of RORE [5′‐(A/G)GGTCA‐3′ or 5′‐TGACC(C/T)‐3′] sites exist at the 5′‐upstream region of the *Gdf10* and *Gdf15* genes (−2939/−2215 for *Gdf10*, and −1240/−922 and −2959/−2707 for *Gdf15*), whereas there are few or no canonical E‐box (5′‐CACGTG‐3′ or 5′‐CACGTT‐3′) sites. In light of our observation of highly expressed *Gdf10 and Gdf15* genes in cultured UESCs treated with the REV‐ERB*α* antagonist, we further analyzed the binding of REV‐ERB*α* to their upstream regions from their transcriptional start sites. The ChIP assay results indicated that REV‐ERB*α* could bind to the RORE sites on the putative promoters of *Gdf10* and *Gdf15* (Fig. 9). We therefore suggest that the expression of *Gdf10* and *Gdf15* genes may be directly under the regulation of REV‐ERB*α* as a repressor by recognizing the RORE sites of the *Gdf10* and *Gdf15* promoters. Both *Gdf10* and *Gdf15* displayed low expression levels in the UESCs of the pregnant D4.5 rats.

Conversely, the expression levels of *Gdf10* and *Gdf15* were significantly increased in the decidual cells. Taken together with these findings, we propose that the clock oscillator silencing may contribute to the upregulation of the *Gdf* gene expression in decidual cells. Thus, REV‐ERB*α* is recruited to the *Gdf* gene promoters in the cultured UESCs and is directly bound to the RORE sites in the *Gdf* gene promoters. Consequently, REV‐ERB*α* acts as a transcriptional silencer to repress the expression of *Gdf10* and *Gdf15* in UESCs.

In conclusion, our experiments demonstrated that the cellular circadian oscillators of rat UESCs regulate the transcription of *Gdf10* and *Gdf15* genes. REV‐ERB*α* can repress the expression of *Gdf10* and *Gdf15* genes by recognizing the RORE sites of the target gene promoters. The attenuation of REV‐ERB*α* may lead to an upregulation of *Gdf* genes in decidual cells, in which cellular oscillators are impaired. This study revealed novel evidence regarding the physiology functions of circadian oscillators regulating the expression of downstream genes during the differentiation of UESCs.

## Conflict of Interest

No conflicts of interest, financial or otherwise, are declared by the authors.
